# Dangerous Driving in a Chinese Sample: Associations with Morningness-Eveningness Preference and Personality

**DOI:** 10.1371/journal.pone.0116717

**Published:** 2015-01-23

**Authors:** Weina Qu, Yan Ge, Yuexin Xiong, Richard Carciofo, Wenguo Zhao, Kan Zhang

**Affiliations:** Key Laboratory of Behavioral Science, Institute of Psychology, Chinese Academy of Sciences, Beijing, China; University of Rome, ITALY

## Abstract

Individual differences in morningness-eveningness preference may influence susceptibility and response to sleepiness. These differences could influence driving performance, but the influence of morningness-eveningness preference on driving behavior and accident risk has not been comprehensively studied. As morningness-eveningness preference is associated with personality characteristics, we also investigated how the interaction between morningness-eveningness preference and personality may be related to dangerous driving behaviors. Two hundred and ninety five drivers completed the reduced Morningness-Eveningness Questionnaire, the Dula Dangerous Driving Index, and personality scales for agreeableness, conscientiousness and neuroticism, and reported demographic information (gender, age, level of education, driving years and annual average driving mileage) and self-reported traffic violations (accidents, penalty points and fines). The results showed that more Risky Driving, Aggressive Driving, Negative Cognitive/Emotional Driving and Drunk Driving, as measured by the Dula Dangerous Driving Index, were all significantly correlated with more eveningness, corresponding to lower scores on the reduced Morningness-Eveningness Questionnaire. Moreover, eveningness was correlated with self-reported traffic accidents, penalty points and fines. Furthermore, a moderation effect was found: eveningness was more strongly associated with risky driving and negative emotional driving in those who scored high for trait agreeableness.

## Introduction

More and more people are dependent on vehicle transportation at all times of the day or night. Previous studies have found that sleepiness plays an important role in highway accidents, and that these accidents are associated with a circadian pattern [1]. Although traffic conditions and environmental factors could explain some aspects of the relationship between time of day and road accidents [2], the morningness-eveningness preference of drivers may also be a potential factor which could influence drivers' behavior [3,4]. However, few studies have investigated the association between morningness-eveningness preference and driving behavior.

The systematic study of morningness-eveningness preference is relatively recent and has developed rapidly in the last two decades. Morningness-eveningness preference can be considered as one of the most marked individual differences in circadian rhythms [5]. Given the individual differences in diurnal preferences, sleep-wake patterns, activity patterns, and alertness in the morning and evening, three categories of circadian typology (CT) can be identified: morning-type (MT), neither-type/neutral-type (NT), and evening-type (ET) [6]. MT people (“Morning larks”) are those who awake and go to bed earlier, and achieve their peak mental and physical performance in the early part of the day. On the other hand, ET (“Night owls”) go to bed and get up later, and perform at their best towards the end of the day [6–8]. NT show patterns of behavior belonging to an intermediate area between the two extreme types [5].

Morningness-Eveningness preference is related to cognitive performance [8]. ET tend to achieve higher levels of performance in working memory tasks than do MT [9]. Furthermore, previous studies have shown a connection between morningness-eveningness preference and driving performance [4]. MT reported better driving ability at 06:00, 08:00, and 10:00, while ET rated their ability better at 22:00 and 00:00 [10]. Another study showed better driving performance (fewer driving errors and code violations) among MT [11]. There are several factors related to the twenty-four-hour pattern of car accidents, including time of day variation. Lenné et al. [12] found that, at 6 a.m. and 2 a.m., the performance of the drivers (the mean and standard deviation of lateral position and speed) was bad; between 10 a.m. and 10 p.m., some improvements were observed, but there also existed a dip in the early afternoon. Another recent study suggests that morningness-eveningness preference plays an important role as a modulator of the relationship between time of day and driving safety. MT had a better performance (i.e., drove more safely) than the ET only in the morning session [13]. There has been little research on the relationship between morningness-eveningness preference and traffic accidents, however, a study about the occupational risk of motor vehicle collisions for emergency medical residents showed no correlation between motor vehicle collisions or near-crashes and morningness-eveningness preference [14].

The relationship between the Morningness-Eveningness dimension and personality traits has been extensively investigated [7, 8, 15]. Research on the Five Factor Model has shown that conscientiousness is positively correlated with morningness, and, furthermore, it is considered the best predictor of morningness [7, 16]. ET score lower for conscientiousness and report psychological disturbances more frequently and intensively than do MT [17]. Conscientiousness is also negatively correlated with risky driving [18], aggressive driving [19], and self-reported accidents [20]. However, some studies have shown no associations between morningness-eveningness preference and extraversion or openness [16, 21], so these traits were not assessed in the current study.

Agreeableness has been found to have a positive correlation with morningness [6, 21], and is negatively correlated with aggressive and risky driving, the number of traffic regulation violations and accidents [22]. Research findings on the relationship between morningness-eveningness and neuroticism are not consistent. A study found that MT reported higher neuroticism scores [23]. However, other studies have found that ET is higher in neuroticism [16, 24]. Findings for the relationship between neuroticism and driving behavior are also inconsistent. Some research has shown that neuroticism is positively correlated with risky driving, aggressive driving [18], and the number of accidents [25, 26]. However, a 34-country study found that neuroticism was negatively linked with traffic fatalities [27].

Until now, no research has explored the interaction effect of morningness-eveningness preference and personality on driving behavior. A study reported that some personality traits and behavioral styles of ET were connected with difficulties of coping with environmental pressure [28]. There are also some individual differences associated with morningness-eveningness preference [15] that are of great importance, such as age [29]. Research in many countries has shown that young children tend to show more morningness, while adolescents show a shift to evening preference, and older people show more morning preference [7, 15, 30]. Young drivers may have more drowsiness in vehicle crashes late at night and in the early morning hours [4].

The main aim of this study was to investigate the relationships between morningness-eveningness preference and dangerous driving behavior. The absence of studies exploring links between morningness-eveningness preference and dangerous driving behavior indicates that this is an area worthy of investigation. Furthermore, morningness-eveningness preference and personality are associated. Although the topic of personality and dangerous driving has been previously investigated, no studies known to us have specifically examined if and how morningness-eveningness preference and personality traits might together be associated with dangerous driving behavior. The relationship between morningness-eveningness preference and dangerous driving behavior could be modulated by specific personality traits. The influences of conscientiousness, agreeableness, and neuroticism were examined.

## Methods

### Participants

Data was collected from 310 licensed Chinese drivers by a research company through interviewing people around parking lots or residential quarters; 295 valid questionnaires were collected. There were 148 males and 147 females, aged from 18 to 55 years old (M = 37.34 years, SD = 9.39). Details of participant demographics are shown in Table 1.

**Table 1:** Participant demographics.

		N	Percent (%)
Age groups by gender	18–30 years		
	Males	41	13.9
	Females	38	12.9
	31–45 years		
	Males	69	23.4
	Females	75	25.4
	46–55 years		
	Males	38	12.9
	Females	34	11.5
rMEQ	Evening-type (4–11 points)	15	5.1
	Neutral-type (12–17 points)	108	36.6
	Morning-type (18–25 points)	172	58.3
Education	Junior high school	18	6.1
	Technical secondary school	28	9.5
	High school	110	37.3
	Junior college	65	22.0
	College or above	74	25.0
Number of years driving	1–3 years	75	25.4
	4–5 years	77	26.1
	6–10 years	71	24.1
	More than 10 years	72	24.4
Annual Average mileage (kilometers)	less than 5000	67	22.7
	5000–10000	145	49.2
	More than 10000	83	28.1
Accident numbers	0	173	58.6
	1–3	122	41.4
Penalty points	0	194	65.8
	1–5	78	26.4
	More than 5	23	7.8
Fines (Chinese Yuan/RMB)	0	160	54.2
	50–200	98	33.2
	More than 200	36	12.2
	Unknown	1	.3

All the participants completed the questionnaire voluntarily and anonymously with a gift given in compensation, and they were told that their information would be strictly confidential and would only be used for scientific research. Ethical approval was given by the Institutional Review Board of the Institute of Psychology, Chinese Academy of Sciences.

### Instruments

**The reduced Morningness-Eveningness Questionnaire (rMEQ).** The five-item reduced Morningness-Eveningness Questionnaire (rMEQ) [30,31] is derived from the Morningness-Eveningness Questionnaire (MEQ) [32], which assesses time of day preference, including items measuring rising time, peak time, sleeping time, morning freshness, and self-evaluation of morningness-eveningness preference. The Chinese version of the rMEQ [30], derived from the Chinese translation of the MEQ [33], was used in the current research, and showed acceptable reliability (α = 0.65). Various cut-off scores for classifying circadian typology have been proposed [30–34], but scale scores can also be used as a continuum [35]. Scores on the rMEQ range 4–25, with higher scores indicating more morning preference. Using the cut-off criteria proposed by Adan and Almirall [31] circadian typology classifications in our study were as follows: 4–11 = evening-type (N = 15/5.1%); 12–17 = neutral-type (N = 108/36.6%); 18–25 = morning-type (N = 172/58.3%); see Table 1. The distribution of rMEQ scores by quartiles was: 63 (21.4%) scored 4–15; 176 (59.6%) scored 16–20; and 56 (19%) scored 21–25. Further analysis in the current study was based on rMEQ scores used as a continuum.

**Personality scales.** As suggested by previous work [6, 7, 16], three personality dimensions were assessed using scales from the Big Five Inventory-44 (BFI-44) [36]: agreeableness (9 items), conscientiousness (9 items) and neuroticism (8 items). Each scale showed acceptable internal consistency with α = 0.73 for agreeableness, α = 0.73 for conscientiousness and α = 0.78 for neuroticism. Each item of each scale was scored on a 5-point Likert scale ranging from “fully disagree” to “fully agree”.

**The Dula Dangerous Driving Index (DDDI).** The Dula Dangerous Driving Index (DDDI) [37] was used to assess the everyday driving behavior of the participants. The Chinese version has previously shown good internal consistency (all subscales α>0.70, except for drunk driving, α = 0.62) and acceptable convergent validity [38]. A total of 28 items, divided into 4 subcategories/subscales, were included. Each item was rated on a 5-point Likert scale, ranging from 1 (never) to 5 (always). Higher scores indicate more dangerous driving behaviors. The total score of the DDDI demonstrated excellent internal consistency (α = 0.92), while the reliabilities of the four subscales were as follows: Risky Driving (RD; 12 items; α = 0.87); Negative Cognitive/Emotional Driving (NCED; 9 items; α = 0.77); Aggressive Driving (AD; 7 items; α = 0.80); and Drunk Driving (DD; 2 items; α = 0.40).

**Demographic questionnaire.** The demographic questionnaire consisted of nine items that requested information related to gender, age, level of education, driving years, annual average driving mileage, number of accidents over the previous three years, penalty points and fines during the past year, including the relevant details (for example, one receives six penalty points when caught driving through a red light, so 6 penalty points, the penalty and the reason why fined should be written down).

## Results

### Descriptive statistics

Table 2 shows mean scores, standard deviations and Cronbach’s alpha for each scale.

**Table 2:** Descriptive statistics for all scales.

Scales	subscales	Mean(SD)	Cronbach’s alpha
rMEQ		17.53(3.27)	.65
The personality scale	Agreeableness	4.13(0.50)	.73
	Conscientiousness	4.07(0.51)	.73
	Neuroticism	1.92(0.63)	.78
DDDI	DDDI total	53.65(16.47)	.92
	NCED	20.24(6.08)	.77
	AD	12.12(4.43)	.80
	RD	18.98(7.63)	.87
	DD	2.31(0.67)	.40
Demographic questionnaire	accidents	0.70(0.95)	
	penalty points	1.18(2.08)	
	fines	125.34(197.11)	

Cronbach’s alpha is for the reliability of each scale.

Note: rMEQ = reduced Morningness-Eveningness Questionnaire; NCED = Negative Cognitive/Emotional Driving; AD = Aggressive Driving; RD = Risky Driving; DD = Drunk Driving; DDDI total = total score for the Dula Dangerous Driving Index.

### Correlation analyses

Pearson correlations between the variables are shown in Table 3. According to Cohen [39], a correlation of 0.5 is large, 0.3 is moderate and 0.1 is small. That is to say, a correlation less than 0.1 is trivial, between 0.1–0.3 is small, between 0.3–0.5 is moderate, and more than 0.5 is large. In the current study, all significant correlation coefficients were greater than 0.1. As shown in Table 3, the negative correlations between rMEQ and the DDDI, including its four subscales, were significant (all *p*<.01). Most coefficients were moderate, indicating that dangerous driving behavior is associated with eveningness. Furthermore, rMEQ was significantly negatively correlated with accident numbers, penalty points and fines (all *p*<.05), indicating some associations between eveningness and traffic violations. The negative relationship between rMEQ and neuroticism was significant (*p*<.01), while rMEQ showed significant positive correlations with agreeableness and conscientiousness (*p*<.01). Agreeableness and conscientiousness were negatively correlated with the DDDI and its subscales (all *p*<.01), while neuroticism was positively correlated with them (all *p*<.01).

**Table 3:** Pearson correlations between variables.

	1	2	3	4	5		6	7	8	9	10	11	12	13	14
1.gender	1														
2.age	−0.028	1													
3.annual mileage	−.215^**^	0.107	1												
4.rMEQ	0.062	.251^**^	−0.064	1											
5.Agreeableness	0.097	.186^**^	−.120^*^	.306^**^	1										
6.Conscientiousness	0.039	.243^**^	−0.091	.370^**^	.752^**^		1								
7.Neuroticism	0.075	−.217^**^	0.014	−.367^**^	−.712^**^		−.748^**^	1							
8.NCED	−.117^*^	−.164^**^	0.047	−.340^**^	−.263^**^		−.345^**^	.382^**^	1						
9.AD	−.188^**^	−.194^**^	.129^*^	−.382^**^	−.445^**^		−.486^**^	.469^**^	.709^**^	1					
10.RD	−0.101	−.198^**^	.147^*^	−.416^**^	−.417^**^		−.480^**^	.542^**^	.737^**^	.652^**^	1				
11.DD	.123^*^	−.214^**^	−0.044	−.276^**^	−.374^**^		−.336^**^	.380^**^	0.091	.317^**^	.253^**^	1	.		
12. total DDDI	−.136^*^	−.213^**^	.119^*^	−.433^**^	−.426^**^		−.494^**^	.534^**^	.905^**^	.846^**^	.921^**^	.277^**^	1		
13.accidents	.177^**^	0.001	0.021	−.145^*^	−.308^**^		−.224^**^	.295^**^	.140^*^	.133^*^	.267^**^	.209^**^	.220^**^	1	
14.penalty points	−.160^**^	−0.051	.308^**^	−.286^**^	−.218^**^		−.272^**^	.172^**^	.151^**^	.271^**^	.268^**^	.143^*^	.258^**^	.200^**^	1
15.fines	−.151^**^	−0.048	.262^**^	−.262^**^	−.176^**^		−.218^**^	0.108	.133^*^	.195^**^	.286^**^	.158^**^	.240^**^	.217^**^	.810^**^

Higher absolute value of coefficient refers to closer relationship between the two variables; +/− is for positive/negative relationship.

Note: All tests are two-tailed. rMEQ = reduced Morningness-Eveningness Questionnaire; NCED = Negative Cognitive/Emotional Driving; AD = Aggressive Driving; RD = Risky Driving; DD = Drunk Driving; Total DDDI = total score for the Dula Dangerous Driving Index.

^*^ *p*<.05

^**^ *p*<.01

Considering the significant correlations between the demographic variables and some of the main variables, we calculated partial correlations, controlling for gender, age and annual mileage. As the significance of the relationships between the variables was not changed for the results in the partial correlations, compared with the original correlation results, we have reported the original coefficients.

### Regression analysis

**Figure 1:**
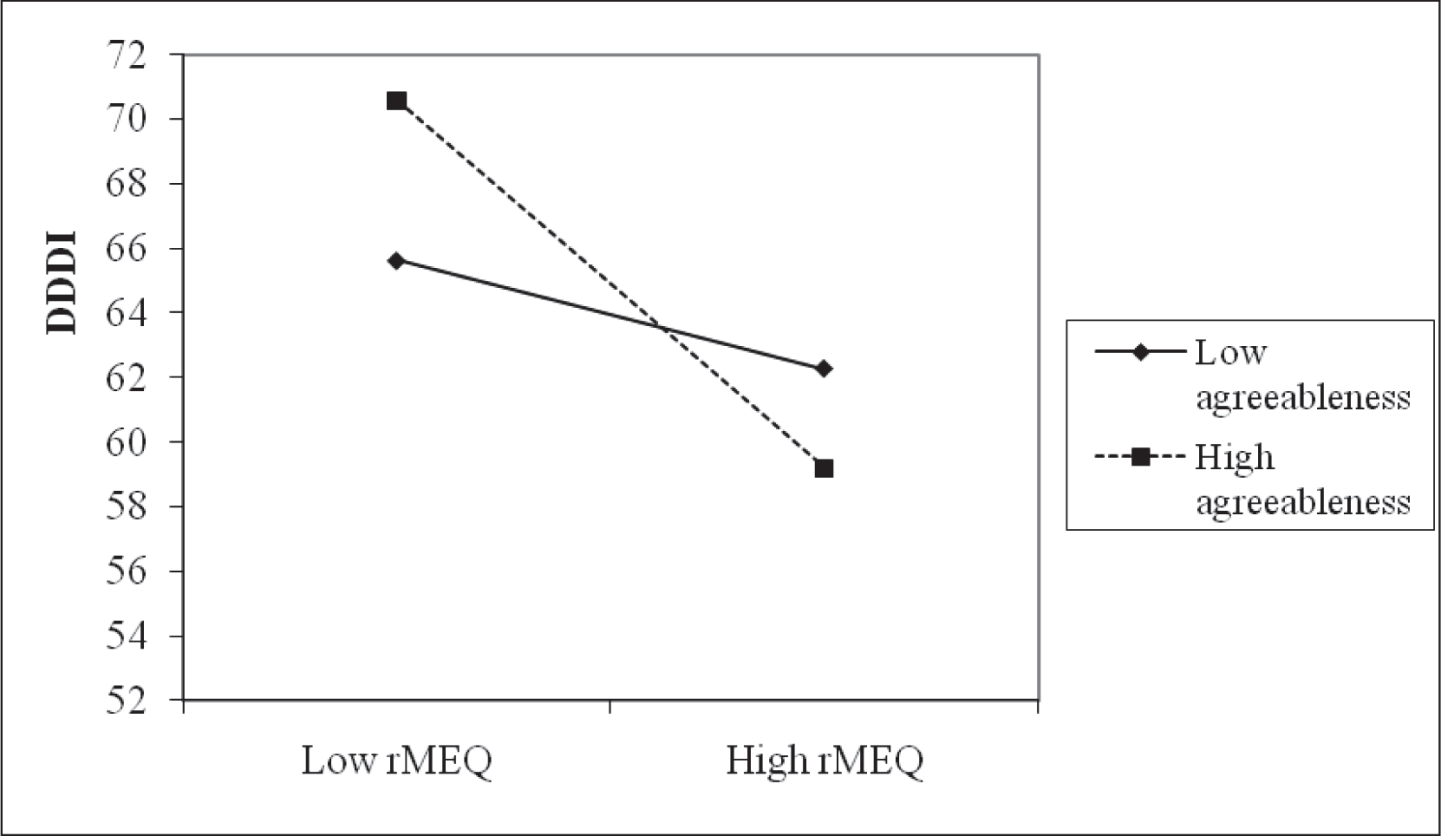
The interaction of rMEQ and agreeableness on DDDI. In the high agreeableness group, drivers with lower rMEQ scores reported significantly higher DDDI than did drivers with higher rMEQ, while no significant result was shown in the low agreeableness group.

Hierarchical multiple regression analysis was conducted to examine the moderating effects of personality traits when regressing dangerous driving behaviors (DDDI, including its four subscales, accidents, penalty points and fines) on rMEQ, controlling for gender, age, and driving years. Following Aiken and West [40], rMEQ and the three personality traits were all mean-centered and interaction scores were also based on the mean-centered scores. Hierarchical multiple regression analysis was conducted in the following steps: gender, age and driving years were entered at step 1; agreeableness, conscientiousness and neuroticism at step 2; rMEQ at step 3; and the two-way interaction of rMEQ and each of the personality traits at step 4. Table 4 shows the detailed results of the regression of DDDI. A surprising result was that there was no significant effect of agreeableness on DDDI, contrary to previous work. However, there was a significant effect of rMEQ on DDDI when controlling for the demographic variables and personality traits. Also, the interaction between rMEQ and agreeableness was significant, indicating that agreeableness moderated the effect of rMEQ on DDDI. The simple slope analyses were tested to examine the nature of the interaction. The results showed that drivers with lower rMEQ scores reported higher DDDI than did drivers with higher rMEQ scores in the high agreeableness group (t = −4.151, *p*<0.001). No significant result was shown in the low agreeableness group; see Fig. 1. The same trends were also found in NCED and RD. To be more specific, drivers in the high agreeableness group, who had lower rMEQ scores got higher NCED (t = −3.139, *p*<0.01) and RD (t = −4.725, *p*<0.001) scores than did those with higher rMEQ scores, while no differences were found in the low agreeableness group for NCED or RD. However, it was opposite for DD: drivers with lower rMEQ scored higher for DD than did those with higher rMEQ, in the low agreeableness group (t = −4.321, *p*<0.001), but not in the high agreeableness group (t = −0.214, *p* = 0.831).

**Table 4:** Results of Hierarchical Regression Analysis on DDDI.

	DDDI			
	Step 1	Step 2	Step 3	Step 4
Gender	−0.13*	−0.16**	−0.14**	−0.13**
Age	−0.26***	−0.13*	−0.08	−0.09
Driving years	0.12*	0.11*	0.09	0.08
Agreeableness		0.03	0.03	−0.003
Conscientiousness		−0.19*	−0.15	−0.15
Neuroticism		0.4***	0.35***	0.33***
rMEQ			−0.22***	−0.29***
rMEQ* Agreeableness				−0.15**
R^2^	0.08	0.35	0.39	0.41
R^2^adj	0.07	0.34	0.37	0.39
R^2^change	0.08	0.27	0.04	0.02
overall F	8.29***	25.77***	26.19***	24.44***
df	3,291	3,288	1,287	1,286

High R^2^ identifies better model; R^2^adj accounts for the number of predictors or traits in the model, the higher, the better; R^2^ change refers to the change of R^2^ compared with that in the previous step.

Note: ^*^ *p*<.05; ^**^ *p*<.01; ^***^ *p*<.001

## Discussion and Conclusion

### Summary of findings

The main contribution of this study is the demonstration that morningness is negatively correlated with dangerous driving behavior. In comparison to ET, MT has been associated with safer driving behaviors [11]. Negative correlations were found between rMEQ score and DDDI-assessed Risky Driving (RD), Aggressive Driving (AD), Negative Cognitive/Emotional Driving (NCED) and Drunk Driving (DD), indicating an association with eveningness. In terms of behavioral style, MT are characterized by high levels of self-control [41] while Wang and Chartrand found that ET are more likely to engage in financially risky behaviors [42]. Furthermore, some research has shown that ET display more levels of impulsiveness [43], sensation seeking [5], and novelty seeking [44], and lower levels of harm avoidance [5] than do MT. To some extent, impulsiveness, sensation seeking, and novelty seeking reflect risk-taking propensity [42]. In terms of cognitive style, a study indicated that ET are more eager to experience immediate pleasure and are disposed to risky behavior, whereas MT are more likely to consider future consequences of their behaviors [45]. Therefore, these findings suggest that ET are more likely to engage in risky behaviors than are MT. In the current study, morningness was also negatively correlated with aggressive driving behavior (e.g., ‘I flash my headlights when I am annoyed by another driver’). Some researchers have suggested that eveningness is related to higher neuroticism and a study has shown that those high in neuroticism show more aggressive driving [26]. In addition, in adolescents, eveningness has also been associated with aggressive behaviors [46, 47]. These results may offer some explanations for the observed correlations between rMEQ scores and aggressive driving behavior in the current study. Also, eveningness (lower rMEQ score) was associated with more negative emotions (e.g., anger, anxiety, frustration and rumination), all of which can occur without aggression but that nonetheless divert attention from driving. Previous studies have found that ET report more anxious/depressive symptoms, and that they have less ability to control irritation and anger than do MT [16]. Also ET tend to report melancholy mood and predisposition to depression [48], whereas MT report greater satisfaction with life [49].

Moreover, we also explored the association between eveningness and traffic accidents and violations. The results showed that rMEQ score was negatively correlated with self-reported traffic accidents, penalty points and fines. Much evidence has shown an association between accidents and time of day. The circadian effect was observed for dozing drivers; about twice as many accidents occur between midnight and 08:00 than in the other 16 hours of the day [50]. Numerous studies demonstrate that performance on a number of tasks deteriorates during the night hours [51]. Moreover, two field studies, one in locomotive operators [52] and the other in long-haul commercial airline pilots [51], which involved both EEG monitoring and performance evaluation, found inattention and performance lapses during night work. Although our research did not investigate the effect of time of day on driving behavior, we did find an association between eveningness and more dangerous driving behavior. As the best performance occurs in line with morningness-eveningness preference-determined expectations [47], future research could explore the interaction between time of day and morningness-eveningness preference on driving behaviors.

Previous studies have shown that agreeableness and conscientiousness are negatively correlated with dangerous driving behavior and that neuroticism is positively correlated with dangerous driving behavior [18]. In the current research, the results of hierarchical regression analyses confirmed the effect of conscientiousness and neuroticism on dangerous driving behavior, but there was no significant influence for agreeableness. However, the interaction between morningness-eveningness preference and agreeableness on dangerous driving behavior was significant. In concrete terms, the influence of morningness-eveningness preference on RD and NECD was different when an individual scored high for agreeableness. When agreeableness was high, eveningness was associated with RD and NCED. When agreeableness was low, the association with eveningness disappeared. Some studies have found that agreeableness predicts risky driving [18]. Moreover, some studies have indicated an association between morningness-eveningness preference and dangerous driving behaviors [3, 4, 13, 42]. However, previous studies only investigated relationships between personality and dangerous driving behavior, or the relationships between morningness-eveningness preference and driving safety. Our results extend the understanding of how morningness-eveningness preference and personality traits are related to the tendency to engage in dangerous driving behaviors. The present study is one of the first to provide evidence for a moderating role of personality in the relationship between morningness-eveningness preference and dangerous driving behaviors.

### Limitations

The current study is limited by its reliance on drivers’ self-reports to measure dangerous driving behavior and morningness-eveningness preference; these reports could be influenced by social desirability. Also, some of the observed correlations, while being significant, were of modest magnitude, indicating that associations with other variables should be explored. In addition, some studies have found that driving performance and accident risk are affected by time of day [12, 13]. Further study could consider the influence of time of day and its interaction with morningness-eveningness preference on dangerous driving behavior and drivers’ performance in a driving simulator. Finally, due to sampling limitations, the participants in the current study are not representative of all drivers in China. Also, there was a skewed distribution of morningness-eveningness preferences in our sample, with a large percentage of morning-type participants and a small percentage of evening-type participants. Consequently, this study only involved dimensional analysis. Future research could enlarge the sample and explore categorical analysis.

## Implications

The present study investigated the interaction effect of morningness-eveningness preference and personality on driving behavior. In light of our results, the joint effect of morningness-eveningness preference and personality should be taken into account in theoretical models of driving behavior. This is a new field of interest in research on driving behavior. This study also has important practical implications for traffic safety and accident prevention. For society and individuals, a better understanding of the joint effect of drivers’ morningness-eveningness preference and personality on dangerous driving, may lead to better management of potentially dangerous driving behavior, and could be used in the selection of professional drivers, including shiftwork drivers.
